# Trastuzumab Exposure in Early Pregnancy for a Young Lady With Locally Invasive Breast Cancer

**DOI:** 10.14740/wjon919w

**Published:** 2015-06-12

**Authors:** Natasha Pianca, Mohsen Shafiei, Mathew George

**Affiliations:** a Department of Medical Oncology, Northwest Regional Cancer Centre, Tamworth, Australia

**Keywords:** Trastuzumab, Breast cancer, Pregnancy

## Abstract

Due to the lack of literature on the effects of trastuzumab in pregnancy, an interest has been taken in a patient that incidentally became pregnant while on adjuvant treatment in the first trimester following diagnosis of locally advanced breast cancer.

## Introduction

Infiltrating ductal carcinoma accounts for 70-80% of invasive breast cancers [1], making it the most common type. It can be classified into three grades depending on its degree of differentiation, with grade 3 being poorly differentiated. Most women present with an abnormal mammogram or a breast mass they have detected themselves [2].

Trastuzumab is indicated for the treatment of patients with HER2-positive localized breast cancer following surgery, as well as with any adjuvant chemotherapy or radiotherapy [3]. HER2 is the human epidermal growth factor receptor which becomes overexpressed in HER2-positive breast cancers. Trastuzumab is a human monoclonal antibody that inhibits the proliferation and survival of such HER2-dependent tumors [4]. In pregnancy, trastuzumab is classed as a category D drug, as it causes embryo-fetal toxicity. Exposure has been known to cause oligohydramnios and oligohydramnios sequence according to the US FDA [5].

## Case Report

The young lady had a previous diagnosis of polycystic ovarian syndrome and was commenced on metformin. She had been trying to become pregnant unsuccessfully in the past and underwent embryo freezing prior to commencing treatment.

Our patient received her diagnosis of grade 2 invasive ductal carcinoma in her left breast (T2N0M0) in 2005, at 30 years of age. It was estrogen and progesterone receptor negative and HER2-positive. The therapeutic wide local excision and axillary dissection demonstrated a 31 mm lesion in the left breast with lymphovascular invasion. She underwent six cycles of adjuvant fluorouracil, epirubicin and cyclophosphamide chemotherapy, followed by left breast irradiation and 4 months of herceptin.

The herceptin was ceased prematurely when she discovered she was pregnant using a urine pregnancy test in late December 2006. USS dating confirmed a viable intra-uterine pregnancy dated at 28/40 weeks. There are a few factors that were retrospectively explored as to why such a mature pregnancy was not picked up earlier, those including: patient body habitus (obesity), as well as amenorrhea secondary to chemotherapy and polycystic ovarian syndrome, and a past history of infertility. Of her seven treatments with herceptin, two of these were thought to have been while pregnant.

Naturally, the patient was monitored throughout her high risk pregnancy by the maternal fetal medicine unit. An USS at 29/40 demonstrated relatively small abdominal circumference and oligohydramnios (AFI 7.7). IUGR was suspected, although no abnormality of the fetus was ever noticed despite there being restricted fetal movements and its continuity of extended breech presentation throughout the pregnancy.

In March 2007, a healthy female was born by an emergency LSCS after SROM and contractions commenced on a breech presentation. The CTG was reassuring after the SROM at 37/40 and Apgars were 4 and 8. Birth weight was 2,735g and baby required minimal resuscitation with O_2_ therapy, suction and IPPR bag and mask. No birth defect was noted. Postnatally, issues breastfeeding after radiotherapy were the only problems encountered and mum seemed to be settling into the role very well, introducing bottle feeds early on. The child, now 7 years old, still does not display any signs of congenital exposure to trastuzumab.

## The Literature

The use of trastuzumab in pregnancy has limited data available, especially in regard to effects on human pregnancies. Until 2010, trastuzumab was listed as a category B drug in the US FDA [5].

Despite the lack of trials, there have been a number of case reports similar to this that describes the outcomes of incidental trastuzumab intake whilst pregnant [6-8]. Most conclude trastuzumab has no immediate adverse effect on the fetus; however, many report the presence of oligohydramnios during pregnancy.

The HERA trial was a large phase III randomized clinical trial in which early HER2-positive breast cancer patients were randomized to receive 1 or 2 years of trastuzumab or observation after chemotherapy [9]. Researchers looked into patients who fell pregnant whilst on therapy that were enrolled in the HERA trial, and found that pregnancy occurring during and up to 3 months after trastuzumab treatment caused larger numbers of spontaneous abortions than the average population (25% patients) and short-term fetal outcomes were normal across all groups being studied. It was also noted that no congenital abnormalities were reported in those patients exposed to trastuzumab *in utero* [10]. This was the first large trial assessing the effects of trastuzumab on human pregnancies and was looking to collaborate data with any other trials to confirm findings.

### Conclusion

The available literature on the effects of trastuzumab on pregnancy in the first trimester is limited. Case reports discuss the presence of oligohydramnios on fetal USS that results in a living baby that seemingly lacks any abnormalities. There may also be a link with higher rates of spontaneous abortion. We describe a young 32-year-old patient on trastuzumab for a locally invasive HER2-positive breast cancer who became pregnant with fetal exposure to the treatment. After cessation of trastuzumab at 28 weeks, she delivered a healthy baby girl at 37 weeks gestation who, at 7 years of age now, still does not display any evidence of negative effects of trastuzumab exposure.
